# Supplementary material for: Identification and characterization of Streptomyces flavogriseus NJ-4 as a novel producer of actinomycin D and holomycin
Source: PeerJ. 2017 Jul 19;5:e3601. doi: 10.7717/peerj.3601 (PMC5520960; doi:10.7717/peerj.3601)
Supplement: Supplemental Information 1 — Figure S1HPLC analysis of the first active compound purification from strain NJ-4. Figure S2 UV analysis of the first active compound isolated from strain NJ-4. Figure S3 Fourier transform infrared (FTIR) spectroscopy of the first active compound purification from strain NJ-4. Figure S4 The structure of actinomycin D. Figure S5HPLC analysis of the second active compound purification from strain NJ-4. Figure S6 UV analysis of the second active compound isolated from strain NJ-4. Figure S7 Fourier transform infrared (FTIR) spectroscopy of the second active compound purification from strain NJ-4. Figure S8 The structure of holomycin. Figure S9 Time course of redox potential of fermentation broth of strain NJ-4 cultured in GS(square) , GSS (circle) and CS (triangle) media. Figure S10 The ratio changes of NAD/NADH of strain NJ-4 cultured in GS (square), GSS (circle) and CS (triangle) media. [file peerj-05-3601-s001.doc]

**
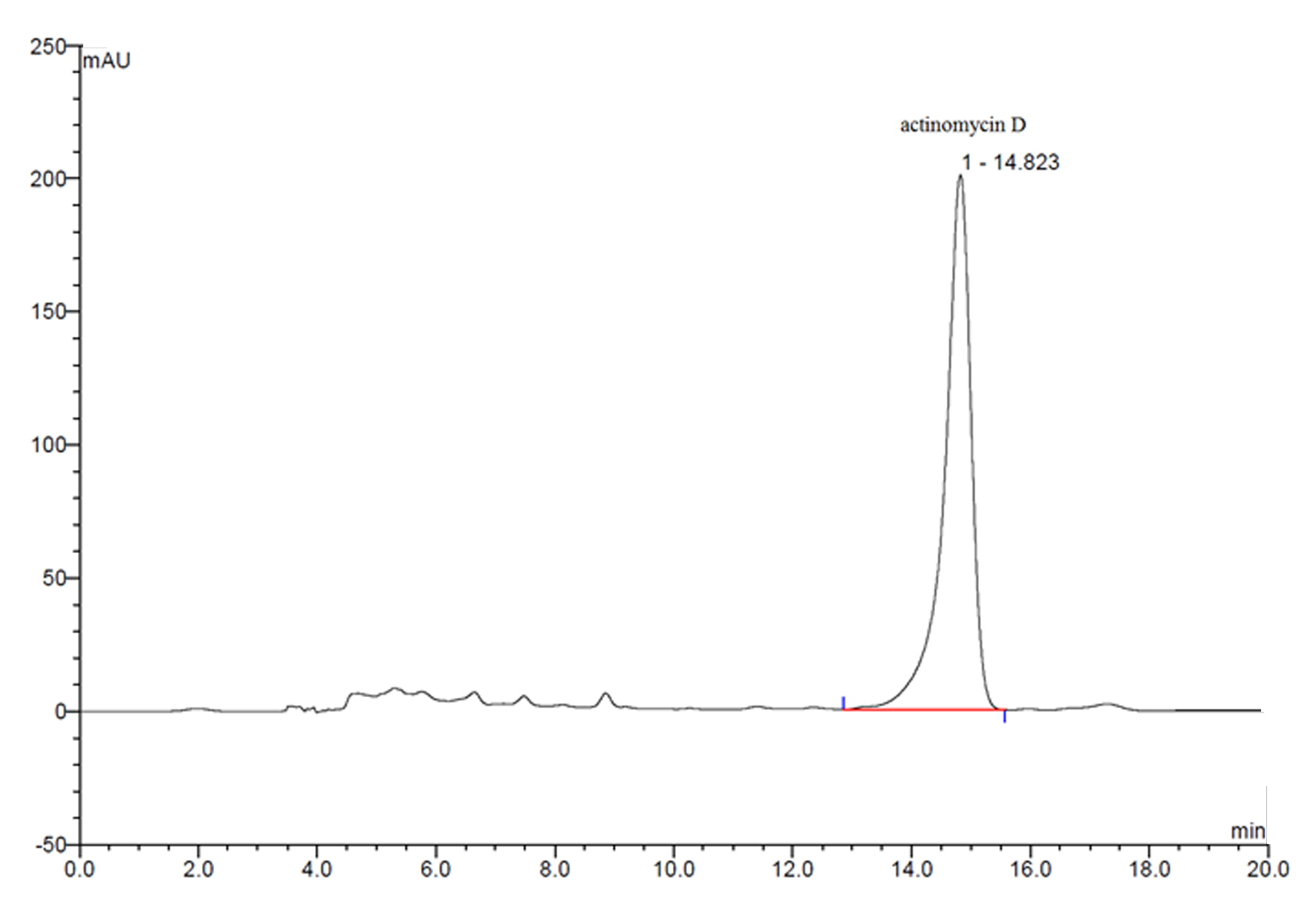
**

**Fig. S1** HPLC analysis of the first active compound purification from strain NJ-4

The first active compound was purified by HPLC with an HPLC retention time at 14.8 min.


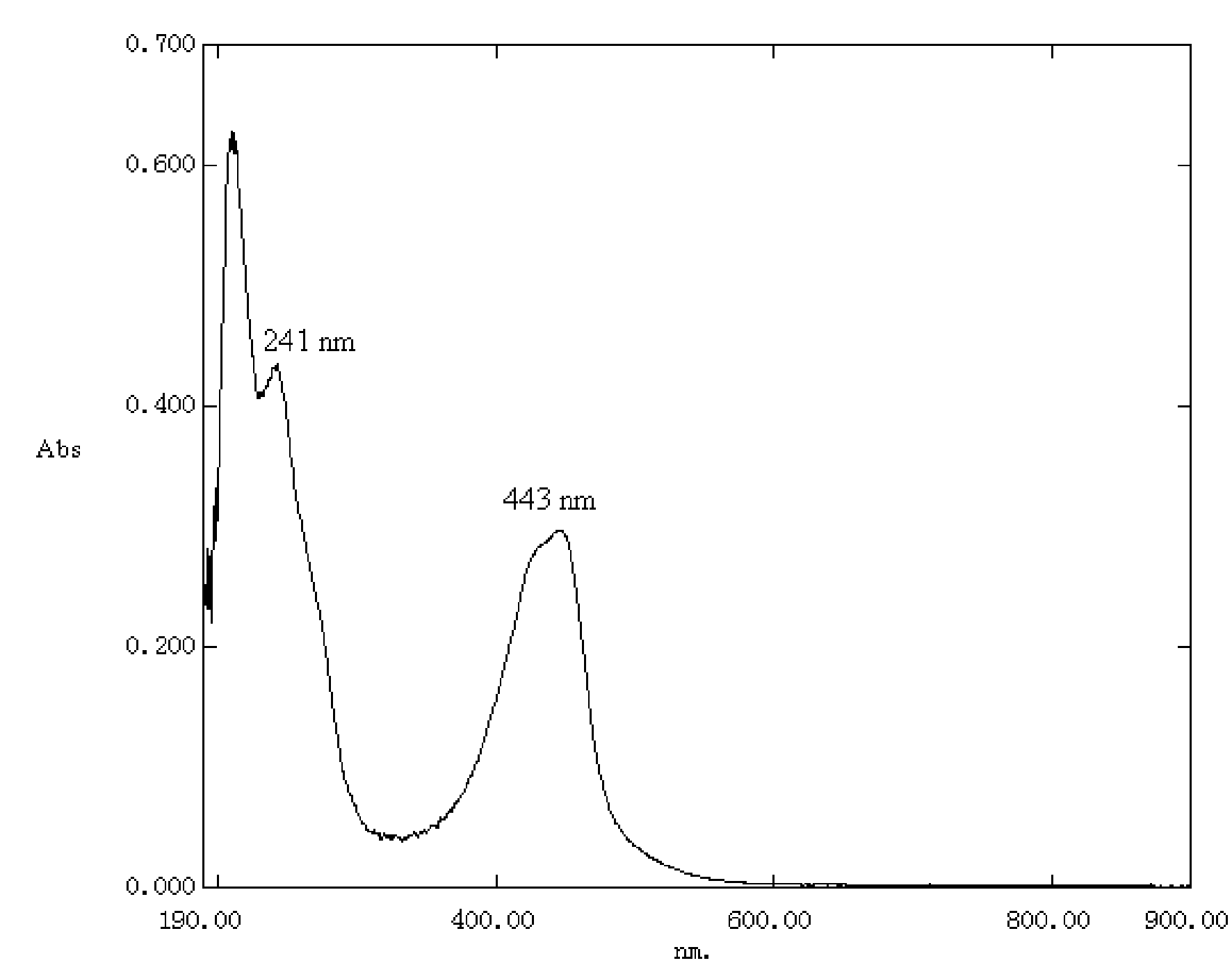


**Fig. S2** UV analysis of the first active compound isolated from strain NJ-4

The first antimicrobial activity component in methanol showed typical UV**-**Vis spectrum with maximal absorbance at 241 nm (shoulder) and 443 nm similar to actinomycin D


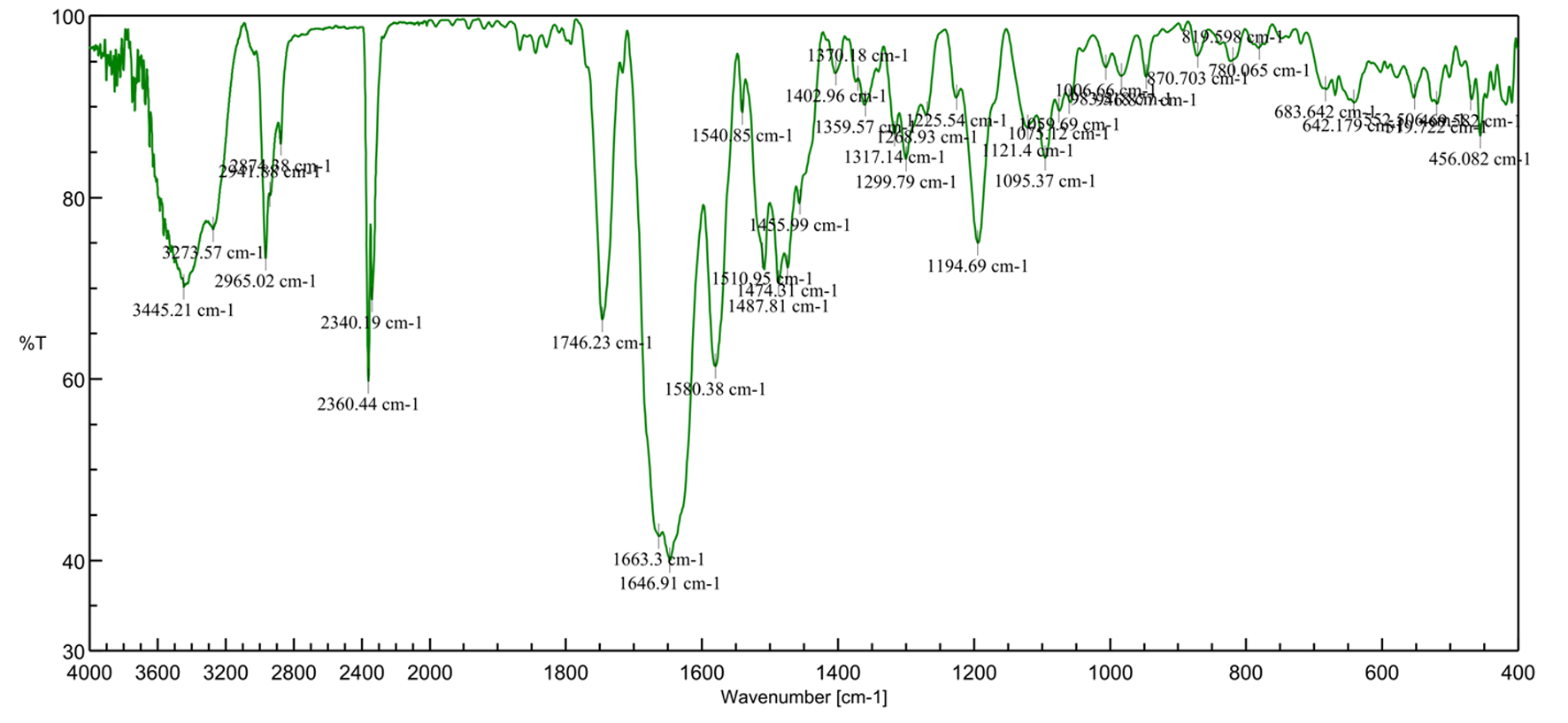


**Fig. S3** Fourier transform infrared (FTIR) spectroscopy of the first active compound purification from strain NJ-4

The IR spectrum (KBr) indicated the presence of -C=O (1746.23 cm^-1^ and 1646.91 cm^-1^) and -NH (3445.21 cm^-1^ and 3273.57 cm^-1^). There were bands at 2874.38 cm^-1^ and 2965.02 cm^-1^ because of the symmetrical and asymmetrical C-H stretching of the -CH2 group.


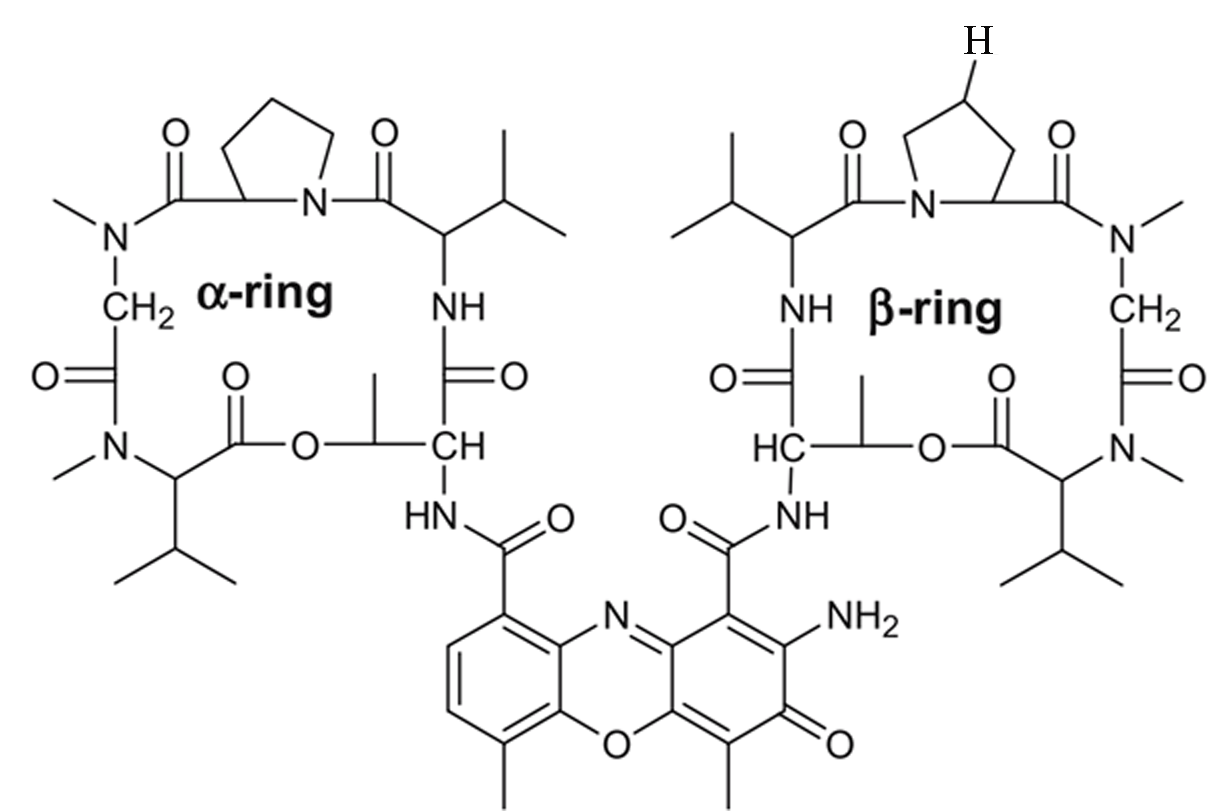


**Fig. S4** The structure of actinomycin D

**
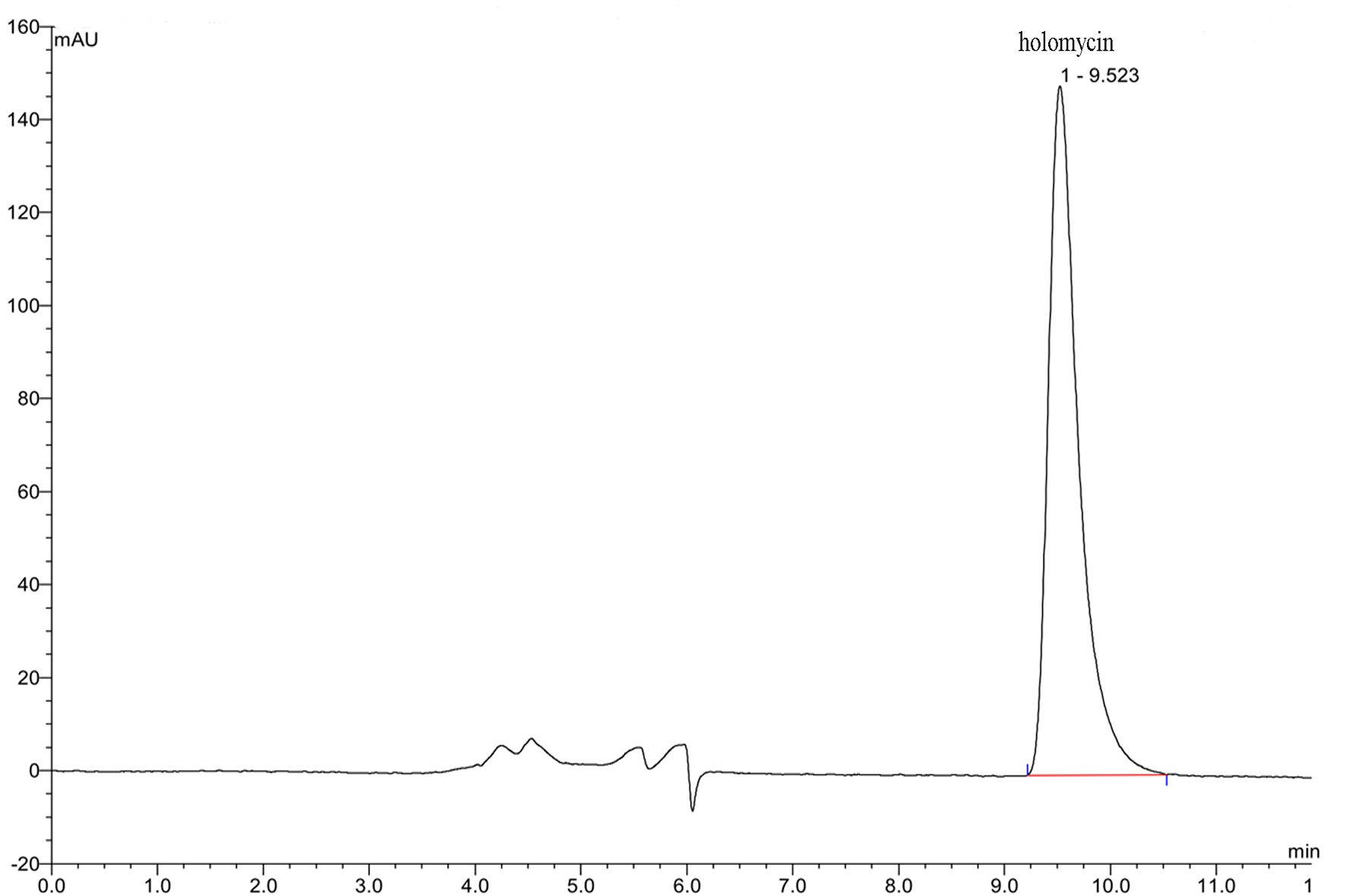
**

**Fig. S5** HPLC analysis of the second active compound purification from strain NJ-4

The second active compound was purified by HPLC with an HPLC retention time at 9.4 min .

**
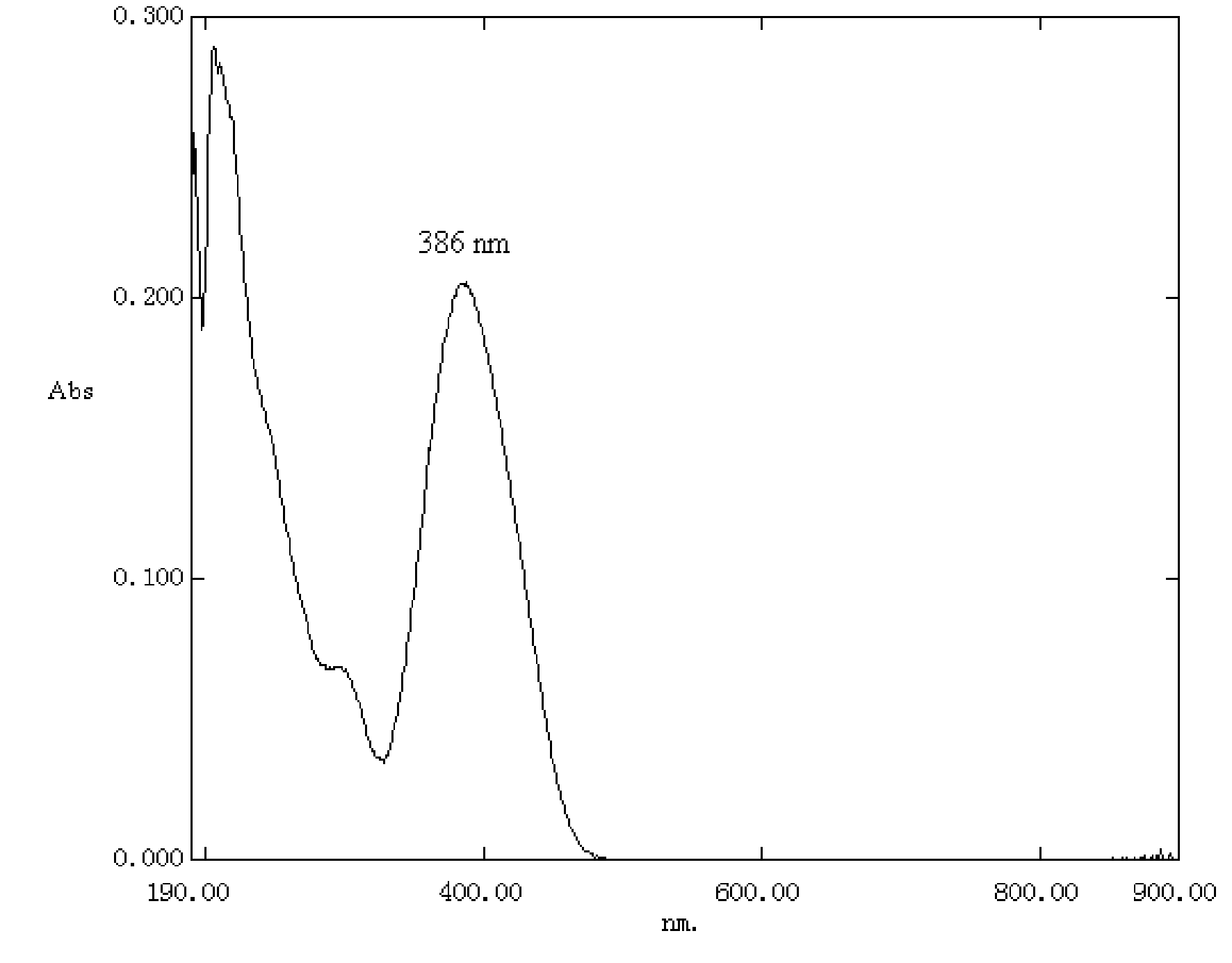
**

**Fig. S6** UV analysis of the second active compound isolated from strain NJ-4

The UV spectrum of the second antimicrobial activity compound in methanol exhibited maximum peaks at 386 nm, which were the trait of pyrroline ring .

**
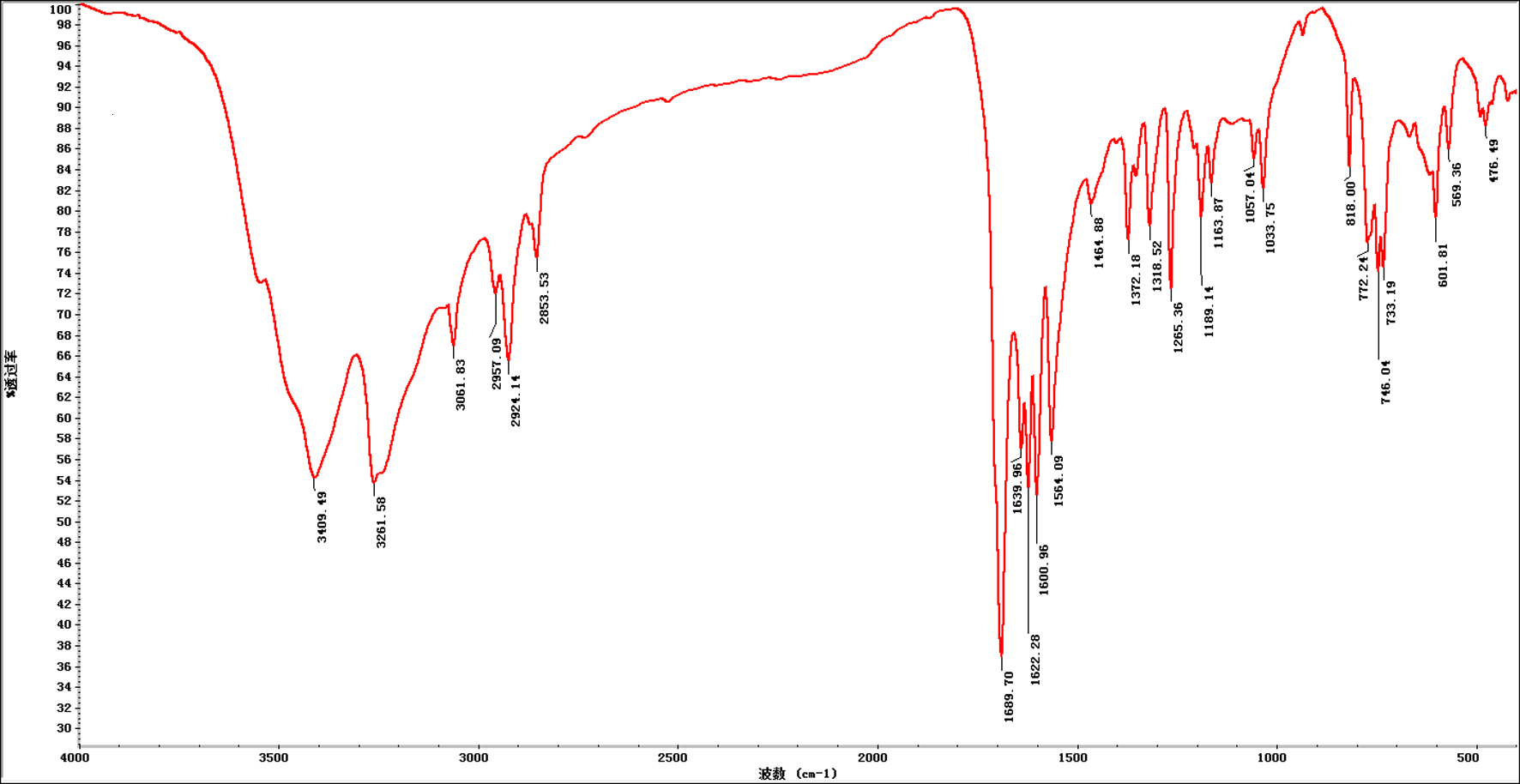
**

**Fig. S7** Fourier transform infrared (FTIR) spectroscopy of the second active compound purification from strain NJ-4

The infrared absorption bands at 3409.49 cm^-1^ and 3261.58 cm^-1^ was due to the stretching vibration of –N**-**H. Bands at 3061.83, 2957.09, 2924.14, 2853.53 cm^-1^ were because of the stretching vibration of –C**-**H. The –C=O stretching vibration was observed at 1689.70 cm^-1^. 1600.96 cm^-1^ and 1564.09 cm^-1^ corresponding to the skeleton vibration of hetero aromatic rings.


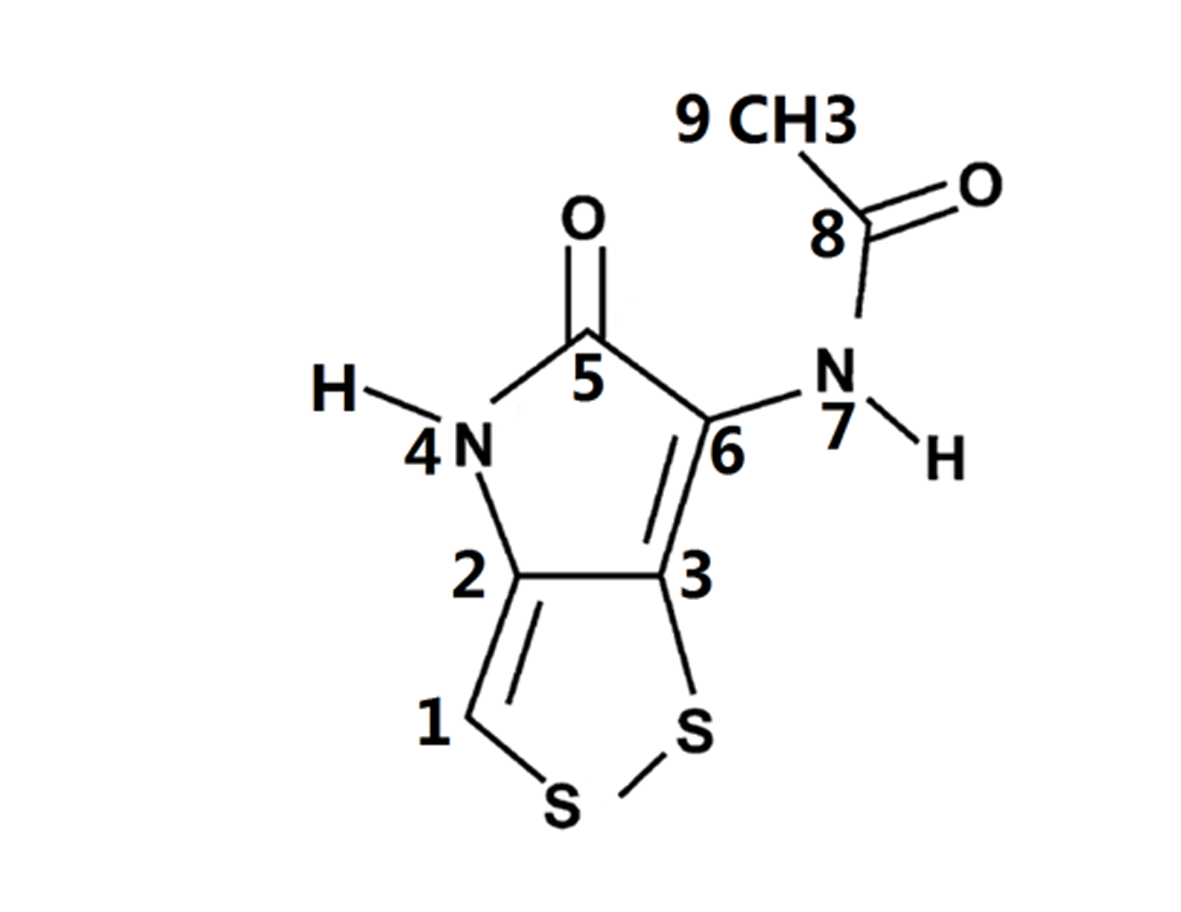


**Fig. S8** The structure of holomycin


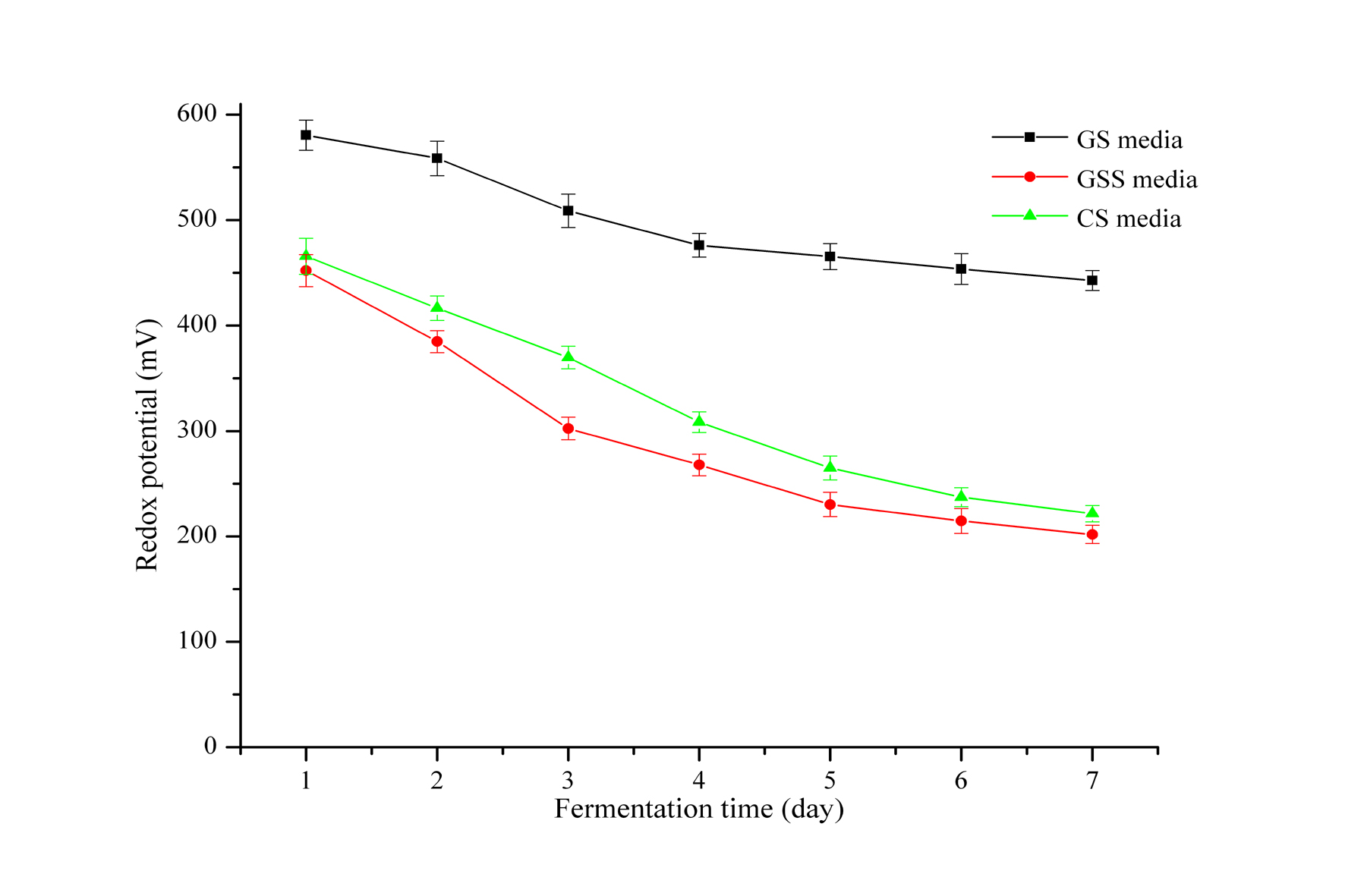


**Fig. S9** Time course of redox potential of fermentation broth of strain NJ-4 cultured in GS(square) , GSS (circle) and CS (triangle) media

In CS medium and GSS medium, soybean flour as an organic nitrogen source reduces the redox level of the fermentation broth.


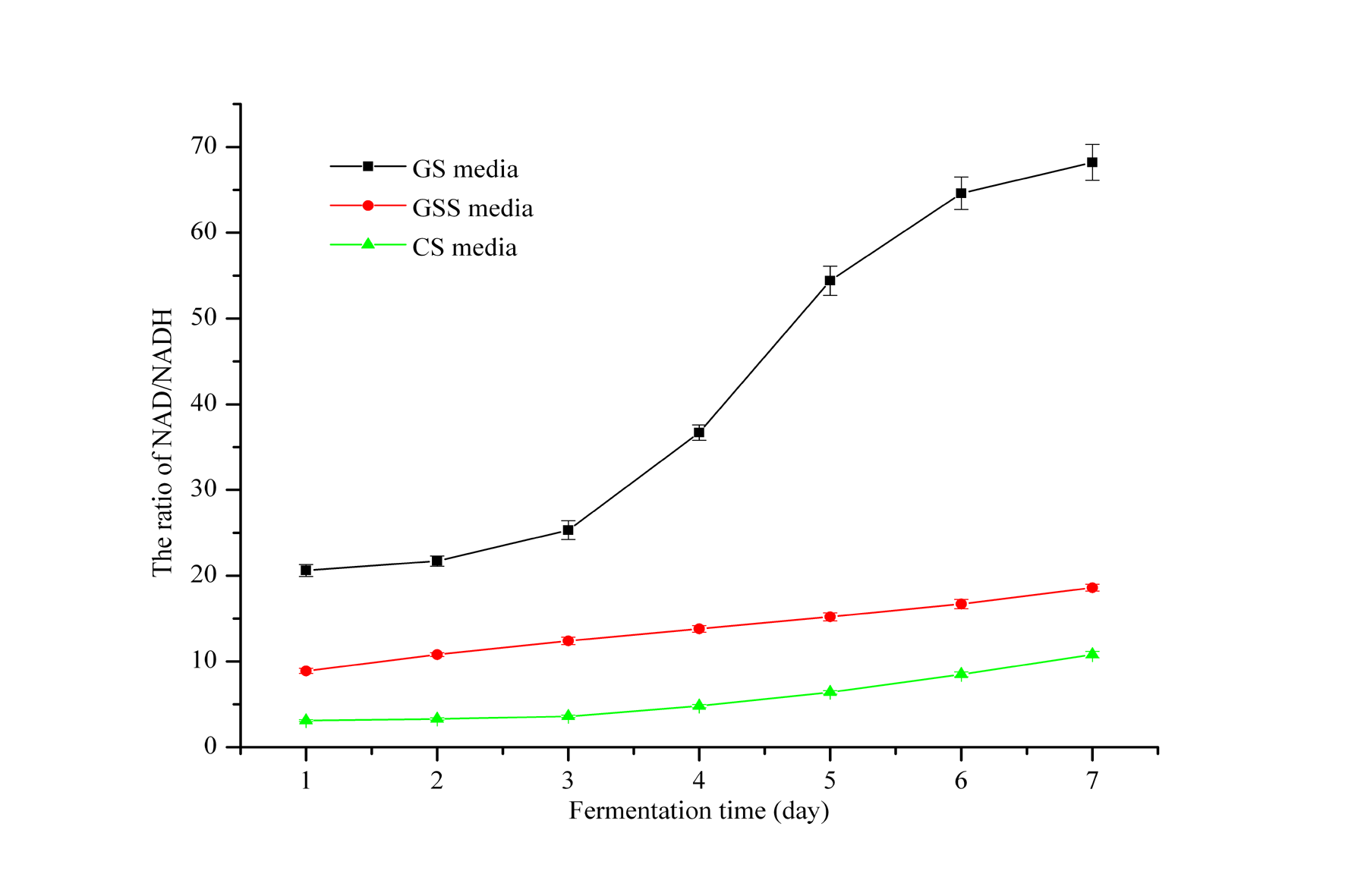


**Fig S10** The ratio changes of NAD/NADH of strain NJ-4 cultured in GS(square) , GSS (circle) and CS (triangle) media

The ratio of NAD/NADH of strain NJ-4 in GS media is greater than in CS medium and GSS medium.
